# Research on the Impact of Outlets’ Experience Marketing and Customer Perceived Value on Tourism Consumption Satisfaction and Loyalty

**DOI:** 10.3389/fpsyg.2022.944070

**Published:** 2022-08-04

**Authors:** Jingyu Dai, Liang Zhao, Qiang Wang, Hailiang Zeng

**Affiliations:** ^1^College of Art Design and Media, Sanda University, Shanghai, China; ^2^College of Humanities, Han-Liang Cultural Research Center, Shangqiu Normal University, Shangqiu, China; ^3^College of Foreign Language and Tourism, Liming Vocational University, Quanzhou, China; ^4^School of Economics and Management, Guangdong University of Petrochemical Technology, Maoming, China

**Keywords:** outlets, luxury marketing, statistical analysis, sustainable development, loyalty

## Abstract

The research object of this subject, through cooperation with Shanghai International Fashion Education Center, a fashion travel education institution, is a convenient sample for the members of its “Japan Fashion Travel Project,” using quantitative research methods and research tools for questionnaires. From the perspective of tourist shopping experience marketing, this paper studies the relationship among tourist marketing, value perception, shopping satisfaction, and customer loyalty to outlets, and discusses the recommendations for sustainable development of outlets.

## Introduction

From the perspective of sales of retail products, especially in the field of luxury goods, manufacturers will encounter such a problem that due to some mistakes some substandard products have appeared. For the luxury goods sector, these products are expensive and maybe just a small mistake that prevents them from providing the noble experience that luxury brands should provide. Out of competition and financial pressure, the prototype of outlets was born. It tends to “broken code promotion, discount promotion,” and other methods, and concentratedly dumps discounted goods in the form of hypermarkets.

With the times of development OR simply says “with this development, outlets have gradually become a new type of industrial model and are closely related to tourism.” Many tour groups will add outlets as one of the means to enrich the tourist experience. In outlet shopping, evaluation systems are mostly related to satisfaction and loyalty. Experiential marketing and perceived value are also related to satisfaction and loyalty.

Reviewing and commenting on the existing literature, we find that most of the existing literature is about the evaluation of sustainable development of department stores. Few people are based on the luxury shopping of outlets. On the other hand, the study found that most of the luxury shopping research occurred in Europe and the United States, and only a little research on the luxury shopping development occurred in Asia. This is also one of the starting points of this paper (Dedeoglu, 2019).

Outlets are an important part of tourism consumption. Italy is an important town of traditional luxury goods, and it is also an area that started the development of outlets tourism retail earlier. The Italian National Tourism Agency (Gunadhi and Boey, 1986) discusses the consumption of foreign tourists in Italy in “On Flow and the Cost of Foreign Tourists Made in Italy.” It uses statistical methods to calculate tourist consumption models, which is the earliest research in this field. It can be seen that outlet shopping is related with tourism consumption.

In 2018, the travel consumers in Wisconsin spent a total of US$170 million on shopping in outlets, accounting for 31% of the total travel expenditures, exceeding the entire volume of transportation and accommodation (Finn and Erdem, 1995). Tourism shopping, as a very attractive item on the journey, has a significant contribution to the destination economy (Jansen-Verbeke, 1991; Cook, 1995; Timothy and Butler, 1995; Di Matteo and Di Matteo, 1996). Many hotels, restaurants, and entertainment venues have gradually appeared as specialized serving outlets (Gunadhi and Boey, 1986).

Outlets have gradually become a new type of industry model and are closely related to tourism. Many tour groups will add outlets as one of the approaches to enrich the tourist experience (Finn and Erdem, 1995). However, the outlets in China are uneven and face developmental difficulties whilst lacking theoretical researches in that field. Therefore, it is urgent and indispensable to do research in regards to outlets (Finn and Erdem, 1995).

The significance of the study is to understand the inner relationship between outlet topic personal experience marketing and customer perceived value, and to explore the influence of outlet personal experience marketing on various dimensions of customer perceived value. Compared with previous studies, this study focuses on outlets’, a special tourism shopping scene, development from the personal and social experiences of tourists (Getz, 2012), and carries out an in-depth quantitative analysis from the psychological perspective of consumers and marketers, and also makes an important contribution in the field of tourism. The purpose of this study is as follows:

First, we hope to understand the internal relationship between experience marketing of outlets and customer perceived value, and on this basis, explore the impact of individual experience of outlets on various dimensions of customer perceived value.
Second, we hope to understand the internal relationship between experience marketing of outlets and customer perceived value. On this basis, we will explore the impact of social experience marketing of outlets on all the dimensions of customer perceived value.
Third, we hope to understand the impact of customer perceived value and customer satisfaction in the context of outlet tourism shopping, to explore the internal relationship between customer perceived value and customer satisfaction with tourism consumption.
Fourth, we hope to discuss the influence and internal relationship between customer satisfaction and customer loyalty in the context of outlet tourism shopping. To explore the differences in tourism consumption between outlets’ consumers with different demographic characteristics.
Fifth, we hope to understand the perceived value of customers as a mediator effect on the relationship between outlet experience marketing and customer satisfaction and loyalty.
  Sixth, we hope to understand both customer perceived value and customer travel consumption satisfaction as a mediator effect on the relationship between Outlet Experience Marketing and customer travel consumption loyalty.

For the above research, we propose the following research questions and propose research hypotheses corresponding to the research questions and research framework.

## Literature Review

Oliver (1980), a Japanese scholar, assumes that consumers will not be satisfied or dissatisfied if the perceived value meets their expectations; consumers will be satisfied if perceived performance exceeds their expectations, and consumers will be dissatisfied if perceived performance is lower than their expectations. All of her hypotheses have been verified. In 1998, Claes Fornell of the National Quality Research Center (“NQRC”) at the Stephen M. Ross School of Business, University of Michigan, applied the structural equation model theory to construct the Sweden Customer Satisfaction Barometer (SCSB) model, and the test showed that consumer satisfaction and consumer loyalty were positively correlated. Pine and Gilmore (2007) pointed out that the evolution of economic value can be divided into four stages, namely product, commodity, service, and experience. The existence of the experience economy provides more conditions for people to enjoy entertainment services, and the economic environment in the outlets provides good conditions for the arrival of the experience economy. Yuan (2016) through the research on the children’s clothing retail market make a point that the action experience is the most critical part of experience marketing, and the perceived value of customers is positively affected. Li’s (2019) research on brand marketing shows that sensory experience marketing is a kind of experience marketing carried out by enterprises, which is moving with sound, pleasing with color, attractive with taste, and moving with emotion. Sensory experience marketing will have a positive impact on the perceived value of customers. Zhang (2019) research on real estate shows that user loyalty is a behavioral variable, which can be interpreted as the intention of reconstruction. Between user loyalty and perceived value, satisfaction can be used as a mediator effect. Moon and Han (2019) and others take THREE SQUIRRELS BRAND as an example to study experience marketing, pointing out that action marketing creates a variety of experiential opportunities for consumers, communicates the core concept of brand and corporate culture connotation to consumers through interaction, and has a positive impact on perceived value (Gunadhi and Boey, 1986).

**FIGURE 1:**
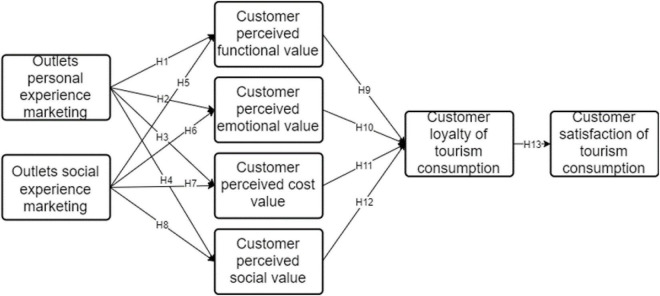
Research model.

Domestic research on experience marketing began in the 1990s, mainly focusing on specific issues under different scenarios, such as clothing store experience marketing, online shopping experience marketing, duty-free store experience marketing, etc. In terms of the dimension division of experience marketing, many domestic studies have conducted case studies at the level of social experience marketing. Jiang et al. (2018) pointed out that enterprises are the main body to successfully implement the experience marketing strategy. They plan the consumer experience process with the arrangement of certain specific scenarios and events as the intermediary, so as to gather consumers to the greatest extent. Liu and Sun (2006) pointed out that after social and economic development and consumption structure optimization to a certain extent, other “economic consumer goods” will be produced, which are not only product consumption in the traditional sense, but also product functions and services obtained during consumption. Hakimi et al. (2018) analyzed the value of experience marketing of offline retail stores from the perspective of online to offline (o2o), and believed that the model of experience marketing can help offline stores attract consumers without fighting a price war with online stores, which is conducive to cultivating customers’ brand loyalty and helping enterprises build the core competitiveness. Given this, this paper mainly analyzes the experience of marketing based on the personal dimension, and build the model (as shown in Figure 1) to make up for the blank of relevant domestic research.

## Model Building

### Research Question 1: The Impact of Outlets’ Personal Experience Marketing on Customer Perceived Value

Through literature review, Schmitt (2004) divides the personal dimensions of experience marketing into sensory, emotional, and thinking. Fang and Li (2006) take the service industry as a sample and divide the perceived value of users into four dimensions: functional value, emotional value, cost value, and social value. Many researchers have concluded that all dimensions of outlets’ personal experience marketing have an impact on customer perceived value.

Wang’s (2015) research based on IKEA marketing means that retailers should add personal emotions to each of the sales when implementing experience marketing, to meet the needs of customers’ psychological and emotional perception value, this has a positive impact on customer perceived value.

Zong (2016), through the study of custom men’s wear experience marketing, said that enterprises through the means of experience marketing enable consumers to deepen their understanding of the brand, and promote their more creative thinking, which has a positive impact on customer perceived social value.

Most scholars have concluded that experiential marketing and perceived value are positively correlated at the individual level. However, each scholar divides the dimension of perceived value differently and lacks the study of outlets’ scenarios.

The outlet’s scene is unique. This research needs to focus on the impact of personal experience marketing on each dimension of perceived value under the outlets’ scenarios.

Based on the above, the following assumptions are proposed in this study:

H1: Outlets’ personal experience marketing has a significant positive impact on customer perceived functional value.

H2: Outlets’ personal experience marketing has a significant positive impact on customer perceived emotional value.

H3: Outlets’ personal experience marketing has a significant positive impact on customer perceived cost value.

H4: Outlets’ personal experience marketing has a significant positive impact on customer perceived social value.

### Research Question 2: The Impact of Outlets’ Social Experience Marketing on Customer Perceived Value

Schmitt (2004) divides social experience into two dimensions: behavior and relevance. Schmitt (2004) divides the personal dimensions of experience marketing into sensory, emotional, and thinking. Li Jianzhou and other scholars (2006) take the service industry as a sample and divide the perceived value of users into four dimensions: functional value, emotional value, cost value, and social value. Through a literature review, the researchers found that many scholars’ research confirmed that the dimensions of outlets’ personal experience marketing have an impact on customer-perceived value.

Liang (2018) believes that, in the study of experience marketing in the tourism industry, once the operation of associated marketing is successful, this part of consumer groups will bring considerable profits to enterprises. Related marketing has a positive correlation with customer perceived value.

Lin (2012) studied the marketing strategy of the hotel service industry and make a point that the fundamental point of the hotel-associated marketing is to effectively carry out experience marketing of sensory, emotional, thinking, and action marketing strategies through the services provided by the hotel, to establish a personal preference for the brand hotel and have a positive impact on the perceived value of customers (Hakimi et al., 2018).

The outlet’s scene is unique. In this study, we need to focus on the impact of personal experience marketing on each dimension of perceived value in outlets. Based on the above, the following assumptions are proposed in this study:

H5: Outlets’ social experience marketing has a significant positive impact on customer perceived functional value.

H6: Outlets’ social experience marketing has a significant positive impact on customer perceived emotional value.

H7: Outlets’ social experience marketing has a significant positive impact on customer perceived cost value.

H8: Outlets’ social experience marketing has a significant positive impact on customer perceived social value.

### Research Question 3: To Explore the Relationship Between Customer Perceived Value and Customer Travel Consumption Satisfaction

To study the influence of customer perceived value and customer satisfaction are the same as to study the influence of different perceived value dimensions of outlets on the improvement of tourist shopper satisfaction.

In 2001, Bai Changhong and Liao Wei pointed out in the gray relation analysis of tourist attractions that customer perceived value is the evaluation and preference formed by customers based on weighing gains and losses against products and services, which has a positive correlation with satisfaction.

Li et al. (2017) pointed out in the study of the service industry that if the demand and expectation of consumers increase, the satisfaction of consumers will decrease. The perceived value of consumers has a positive correlation with consumer satisfaction.

Based on Oliver’s theoretical model, many researchers have made an in-depth definition of tourist satisfaction. The conclusion is drawn that if the performance meets expectations, the tourists are satisfied and *vice versa*. Perceived value is positively correlated with satisfaction.

Dedeoglu (2019) pointed out that service quality, satisfaction, and service value have a direct impact on behavior intention.

Na (2017) pointed out that customer perceived value has a significant impact on consumer satisfaction and purchase intention.

Cheng et al. (2014) showed that the comprehensive evaluation of emotional value, social value, exploratory value, and functional value had a significant positive impact on satisfaction.

The above kinds of literature have discussed the relationship between different dimensions of perceived value and satisfaction in different scenarios but lack the research on a complete dimension of perceived value in the outlets’ scenarios. Based on the above, the following assumptions are proposed in this study:

H9: Customer perceived functional value has a significant positive impact on customer satisfaction with tourism consumption.

H10: Customer perceived emotional value has a significant positive impact on customer satisfaction with tourism consumption.

H11: Customer perceived cost emotional value has a significant positive impact on customer satisfaction with tourism consumption.

H12: Customer perceived social–emotional value has a significant positive impact on customer satisfaction with tourism consumption.

### Research Question 4: To Explore the Impact of Customer Travel Consumption Satisfaction and Customer Travel Consumption Loyalty in the Outlets’ Travel Shopping Scenario

In the literature review, many studies have confirmed that there is a significant correlation between customer travel consumption satisfaction and customer travel consumption loyalty.

Cardozo’s (1965) research results on the retail market show that as long as the consumer satisfaction is improved, consumers will have repurchase ideas (consumer loyalty), which has a significant positive correlation.

In 1999, the European Quality Organization (EOQ), European Foundation for Quality Management (EFQM), and others, along with 12 EU member states, proposed an European Customer Satisfaction Index (ECSI) model that showed a positive correlation between customer satisfaction and customer loyalty.

China Enterprise Research Center of Tsinghua University puts forward the basic model of China’s customer satisfaction index based on China national conditions and points out that satisfaction is positively related to loyalty.

Griffin, a marketing expert, pointed out in 1995 that customer satisfaction and customer loyalty are effective means for companies to maintain their competitive advantages, and the two have a positive correlation.

However, some scholars still point out that sometimes there is no high correlation between satisfaction data and corporate performance, and customers will be more satisfied if they buy elsewhere (Jin et al., 2015).

The existing theory is based on different industry perspectives, and this paper proposes the following assumptions based on outlets tourism shopping:

H13: Customer satisfaction with tourism consumption has a significant positive impact on customer loyalty.

### Research Question 5: The Impact of Customer Perceived Value as a Mediator Effect on Outlets’ Experience Marketing, Customer Satisfaction, and Loyalty

According to the features of the model, a certain mesomeric effect will be formed. In the study of relevant literature on the mesomeric effect, the researcher found that many scholars believe that perceived value can be used as a mediator of experiential marketing, satisfaction, and loyalty (Heung and Qu, 1998).

Harvard professor Porter and Millar (1985) proposed the competitive advantage theory for the retail service industry. He believed that perceived value can greatly enhance the efficiency of marketing between experience marketing and satisfaction, which has a certain positive correlation mediator effect.

According to the consumer behavior model proposed by Brakus et al. (2009), the research shows that brand experience, directly and indirectly, affects consumer satisfaction and loyalty through brand personality, and also affects experience marketing to a certain extent, which has a positive correlation.

Xu’s (2015) research on the influence of perceived value and behavioral intention of budget hotels pointed out that the perceived value of customers has a significant impact on the behavioral intention of consumers and the design of marketing means by managers. Ye et al. (2014) showed that perceived value has a significant effect on increasing barriers to transfer and attitude loyalty, and perceived value has a positive correlation with attitude and loyalty.

Gallarza et al. (2002) proposed a loyalty model. As a mediator effect, perceived value has a positive impact on experience marketing and loyalty.

Wang (2015) and Li (2016), respectively, researched the tourism industry and retail brand IKEA. Their research shows that emotional experience marketing will increase the awareness of the product by increasing the value of emotional perception, thereby increasing the customer’s dependence and satisfaction with the brand, emotion perception value has a significant positive correlation mediator effect.

Based on the above, the following assumptions are proposed in this study:

H14: Customer satisfaction with tourism consumption and customer perceived functional value have a chain-mediated effect between customer loyalty and tourism consumption and personal experience marketing.

H15: Customer satisfaction with tourism consumption and customer perceived emotional value have a chain-mediated effect between customer loyalty with tourism consumption and personal experience marketing.

H16: Customer satisfaction with tourism consumption and customer perceived cost value have a chain-mediated effect between customer loyalty with tourism consumption and personal experience marketing.

H17: Customer satisfaction with tourism consumption and customer perceived social value have a chain-mediated effect between customer loyalty with tourism consumption and personal experience marketing.

### Research Question 6: The Relationship Between Customer Perceived Value and Customer Satisfaction With Tourism Consumption, as a Mediator Effect, and the Impact on Outlets’ Experience Marketing and Customer Loyalty With Tourism Consumption

In the literature review, many studies have explored the relationship between customer travel consumption satisfaction as a mediator effect on experience marketing, perceived value, and customer travel consumption loyalty (Jiang et al., 2018). However, less research has focused on how perceived value and customer travel consumption satisfaction can both serve as a mediator effect, which has a mediator effect on experience marketing and loyalty. Therefore, this study explores the relationship between the above four.

When the customer’s value in consumption increases, his satisfaction will increase and his loyalty to the enterprise will also increase (Heung and Qu, 1998).

Walter et al. (2013) found that the impact of brand experience on satisfaction is not statistically significant, ignoring the link between brand experience and customer perceived value, that is, perceived value is an important moderating variable among customer loyalty, perception, or internal reaction.

The research of Johnson et al. (2003) focuses on tourists who came to Hong Kong from 2000 to 2003. The results show that satisfaction, as a mediator effect, has a positive impact on perceived value and loyalty.

Jiang and Meng (2019) took the platform of the *“ELEME”* software as an example. Taste sensory experience (perceived emotional value) has a certain positive marketing effect on repurchase intention (loyalty), and also has a certain mediator influence on satisfaction.

Getz (2012) studied the perceived value of domestic tourists at Shanghai World Expo. In her research, she pointed out that tourists with different motivations have significant differences in the perceived value of tourism. Her research also pointed out that perceived value has a mediator role in satisfaction and tourism motivation.

According to Petrick et al. (2001), tourism experience, perceived value, satisfaction, revisited tourism, and destination intention have positive a correlation with tourism loyalty, and perceived value and satisfaction have a positive correlation mediating effect.

According to Yuan (2016), satisfaction, as a mediate variable, is positively correlated with loyalty and action experience in social experience marketing.

Many researchers indirectly show that perceived value and customer satisfaction with tourism consumption have a positive correlation with experience marketing and customer loyalty with tourism consumption (Jin et al., 2015). Because the samples come from various industries in the tourism field, there are few studies under the outlets’ scenarios. Based on the above, the following hypotheses are proposed in this study:

H18: Customer satisfaction with tourism consumption and customer perceived functional value play a chain-mediated role between customer loyalty with tourism consumption and outlets’ social experience marketing.

H19: Customer satisfaction with tourism consumption and customer perceived emotional value play a chain-mediated role between customer loyalty with tourism consumption and Outlets social experience marketing.

H20: Customer satisfaction with tourism consumption and customer perceived cost value play a chain-mediated role between customer loyalty with tourism consumption and outlets’ social experience marketing.

H21: Customer satisfaction with tourism consumption and customer perceived social value play a chain-mediated role between customer loyalty with tourism consumption and outlets’ social experience marketing.

Based on the hypothesis, the following model is obtained.

This research is combined with the tourism product “Japan Fashion Week Tour” of Shanghai International Fashion Education Centre (SIFEC). Among the tourism products, there are itineraries for shopping at representative outlets in various places. There are about 20 members per session. The members who participate in the product all have experience in outlet shopping. The researcher selects the top-quality group within 2 years, and ensures with the head of the tour that the users participating in the questionnaire had traveled to outlets for shopping, excluding the groups who have not entered outlets for personal reasons, conducted convenient sampling on the rest of the population, analyzed them as samples and released the questionnaire (Johnson et al., 2003).

The main research method is quantitative research. The study revised the initial questionnaire using the self-developed *“Outlets luxury purchase intention empirical research questionnaire,”* and the researchers invited luxury management experts to conduct in-depth interviews, hoping to determine the initial question items of the questionnaire based on the interview content. The interviewee experts are as Table 1.

**TABLE 1:** Identity of interviewee expert^a^.

1	Chanel’s China Marketing Manager
2	Founder of a popular overseas travel APP called “Jessica’s secret”
3	International EMBA lecturer in luxury goods at Hautes Etudes Commerciales Paris
4	Associate Professor, ITC department, The Hong Kong Polytechnic University

*^a^ Omitting names for privacy reasons.*

According to the time arrangement of the respondents, the researchers conducted five interviews from 13 May to 20 June 2019. The content of the interview provides a scientific and effective basis for the development of the item scale and provides a better supplement for the researchers to understand the connotation of outlets’ experiential marketing, perceived value, satisfaction, and loyalty. By summing up, summarizing, and analyzing the expert opinions, a preliminary questionnaire is formed.

Then, from 30 June to 1 August 2019, the researcher used the method of face-to-face scanning QR code to entrust the overseas study tour Department of SIFEC, aiming at the students of its product “Japanese Fashion Study Tour Project,” to pre-survey by convenience sampling method.

In the pre-survey section, 110 questionnaires were distributed and 102 were recovered. The incomplete and random questionnaires were eliminated, and 93 valid questionnaires were finally obtained for analysis, with a recovery rate of 100% and an effective rate of 97.1%.

Finally, this study uses the SPSS 23.0 software to analyze the items, validity, and reliability of the effective pre-survey questionnaire, to delete the unqualified items. Based on modifying, merging, and again, the formal questionnaire was formed.

Due to the influence of luxury people’s behavior and habits, as well as the influence of the scale of boutique travel groups, the formal research sample is limited to 500, which is one of the research limitations.

## Data Description

After the completion of the pre-test, through the reliability and validity analysis, it is found that the reliability and validity of the pre-test questionnaire meet expectations, and it can be used as a data collection tool for this study. The researchers conducted a formal questionnaire survey from August 2019 to December 2019.

In this study, a total of 460 questionnaires were distributed and 449 were recovered, with a total recovery rate of 97.6%. SPSS 23.0 and Amos 24.0 are used to analyze the empirical data of the questionnaire content, including the following aspects: first, descriptive statistical analysis; second, a test of the measurement model and structural model; third, test the hypothesis proposed in this study; fourth, analysis of variance.

### Descriptive Statistical Analysis

According to the results of descriptive statistical analysis, the average value of each item is above 3.8, and the average scores of all dimensions are relatively consistent, all of which are around 3.95. It can be seen that when consumers consume in outlets, they have requirements for all aspects of perception; among them, the purchase intention of customer perceived functional value is the highest, which is 3.990; second, the average purchase intention of customer perceived cost value is the second highest, which is 3.978; the loyalty is the lowest, which is 3.918, which indicates that consumers have more shopping choices and that outlets are less irreplaceable.

### Subsection Test of the Measurement Model and Structural Model

In this study, the maximum likelihood method was used for confirmatory factor analysis and structural equation model test to estimate parameters, and a prerequisite for this method was that the sample data should be normally distributed.

Therefore, the skewness and kurtosis of all items were tested in this study, and the results showed that the skewness and kurtosis of all items in the questionnaire were within the required range, and accorded with the normal distribution (Kim and Park, 2017), which can be further analyzed. In this study, the Harman single-factor method was used to test the results. The first explanation was 46.830%, no more than 50%. Therefore, the Common Method Variance (CMV) of this questionnaire was not serious.

According to the correlation analysis, there is no significant correlation between personal experience and other variables, but every two of social experience, functional value, emotional value, cost value, social value, satisfaction, and loyalty are significantly positive correlations.

After the analysis and test of reliability and validity, it is proved that the data has corresponding validity and reliability.

The model was fitted by the statistical software AMOS 24.0 using structural equation modeling. It can be seen from the Table 2 below that the dimensions of the service quality fit well. χ^2^ = 1571.745, *df* = 615, and χ^2^/*df* is 2.556. The goodness of fit (GFI) is 0.832, the adjusted goodness-of-fit index (AGFI) is 0.808, the comparative fit index (CFI) is 0.895, and the root means a square error of approximation (RMSEA) is 0.059, indicating that the model fits well.

**TABLE 2:** Conclusion of structural equation model.

			path coefficient	Standard error	T	P	Whether the hypothesis holds
Functional value	<–	Personal experience	–0.020	0.051	–0.400	0.689	No
Social value	<–	Personal experience	0.424	0.078	5.448	***	Yes
Emotional value	<–	Personal experience	0.446	0.070	6.373	***	Yes
Cost value	<–	Personal experience	0.338	0.064	5.288	***	Yes
Functional value	<–	Social experience	0.992	0.083	11.965	***	Yes
Social value	<–	Social experience	0.015	0.085	0.173	0.863	No
Emotional value	<–	Social experience	–0.017	0.074	–0.225	0.822	No
Cost value	<–	Social experience	–0.014	0.068	–0.205	0.837	No
Satisfaction	<–	Functional value	0.046	0.040	1.147	0.251	No
Satisfaction	<–	Social value	0.299	0.049	6.053	***	Yes
Satisfaction	<–	Emotional value	0.465	0.063	7.443	***	Yes
Satisfaction	<–	Cost value	0.295	0.062	4.773	***	Yes
Loyalty	<–	Satisfaction	0.727	0.066	11.049	***	Yes

***< 0.001.

## Conclusion

Through researches on outlets’ shopping trips, some scholars have found that customers perceived value positively affects behavioral intentions (Chen and Chen, 2010). Cost performance is the focus of the research. However, this study found that the customer perceived emotional value is the key to determine the purchase behavior. However, there is a need to point out that the research cases between this study and previous studies from other scholars are quite different. The outlets’ shoppers are a stronger purchasing power compared with other travel shopping types. It is recognized that the outlets’ shopping tourism depends on customers’ emotional perceptions to a large extent. Finally, the results of this study have shown that it is worthy of much more concerns and researches in the tourism industry.

### Outstanding Problems of Current Outlets’ Marketing Strategy

Existing outlets’ marketing strategies lead to low loyalty and high liquidity. Loyalty and perceived social value are relatively low among the existing outlets’ consumers’ shopping intentions. The average loyalty is 3.92, which is the lowest. Among outlet shoppers, fewer are willing to share recommendations and have high loyalty to the brand.

(1) The weak cognitive ability of social status

In terms of social cognitive status, the total mean value of the four items is only 3.93, which is the lower of each item on the scale. Consumers pay more attention to the products and brands of outlets but influenced by the corporate image of outlets and the consistent external discount route, its social status display function is weak.

(2) Adverse selection of consumer groups

Due to the openness and competition of the shopping market, and the positioning of the selling point as discount promotion, it will bring the risk of adverse selection of attracting price-sensitive customers, which is easy to lose and change customers, resulting in relatively low customer loyalty of outlets.

### Significant Differences in Each Path

Outlets’ personal experience marketing and social experience marketing function path is quite different. Personal experience marketing has a more diversified impact, while social experience marketing has a significant impact on functional value. The influence paths of customer satisfaction and customer loyalty are affected by different functions. In customer functional value, social value, emotional value, and cost value have a significant positive influence on customer satisfaction. At the same time, satisfaction has a significant positive impact on customer loyalty.

(1) Outlets’ personal experience marketing is very important, especially in terms of emotion and cost. The relationship between perceived emotional value and personal marketing is the most critical in outlets’ development.
(2) Outlets’ social experience marketing affects customer perceived value mainly in functional value. Social experience marketing, marked by action and relevance, cannot contribute to customers’ perceived emotions, costs, and social values.
(3) The effect of customer perceived function value is weak. In the area of outlets, it is difficult for functional value to be reflected in marketing means, and also hard to improve customer satisfaction.
(4) Customer satisfaction can effectively affect customer loyalty in the context of outlets.

## Research Suggestions

With the rapid development of the outlets’ industry, market competition becomes more and more fierce. To create differentiation and gain the greatest competitive advantage, outlets’ managers need to constantly improve their products and services, and based on improvement, they need to take the satisfaction of tourism retail consumers as the purpose and explore new ways. To provide better services for outlets’ tourism retail consumers and enhance the loyalty of tourism retail consumers.

Based on reviewing and commenting on the relevant literature, this study validates the research hypothesis and concludes. According to the results of the analysis, we can see that outlets have four typical consumer groups, and should adopt a targeted marketing strategy. This study integrates the results of the analysis and puts forward suggestions from two aspects: outlets’ sales personnel and outlets’ managers.

### Suggestions for Outlets’ Sales

(1) Take diversification as the orientation, pay attention to the quality of outlets’ sales staff

In the process of service, the role of sales personnel is extremely important, and ultimately determines the consumption experience of some retail tourism consumers. Sales staff with high professional skills and a high sense of responsibility is an important asset for outlets’ managers (Kent et al., 1983). Outlets are not the distribution center of fast-selling brands, more discounted products of luxury goods are the main sales force. This special attribute is doomed to be a huge difference between it and fast-selling brands.

According to the analysis of relevant data, it can be seen that emotional experience marketing has a significant relationship with the shopping satisfaction of travel retail consumers (Kim and Park, 2017). Outlets’ sales personnel need to follow the consistent principle of luxury sales personnel and give people the luxury flagship store in the service of treating tourism retail consumers. This allows travel retail consumers to maintain the cost-effective advantages of outlet shopping.

In the service process, the employees, especially the retail sales personnel, are the most exposed group with the tourism retail consumers. A good service atmosphere plays an important role in improving the service quality, and also in improving the brand image of outlets’ luxury goods.

Do not differentiate the sales personnel of outlets from those of normal brand stores, and avoid the self-service shopping behavior of travel retail consumers (Kim et al., 2011). The operators of outlets need to set a certain mechanism for supervision, and the luxury brands need to set a certain regional employee rotation mechanism to maintain the consistency of outlets’ brand experience marketing.

The data shows that for some functional products, social experience marketing is very necessary. The salesperson must be familiar with the use of the product, the history of the product, and the explanation of the brand culture, and show them on time. On the other hand, because most outlets’ tourists come from all over the world, it puts forward higher requirements for the language ability and reception ability of salesmen and salesmen (Moon and Han, 2019). Both luxury goods operators and outlets’ managers need to pay attention to the important role of salesmen in the service of tourism retail consumers, which is also the top priority of marketing strategies.

(2) Establish a good service relationship and improve the experience of marketing

In the process of receiving service, tourism retail consumers will form experience. From the perspective of the hypothesis, personal experience will have a significant impact on final consumption, which is stronger than social experience. In the process of service contact with tourism retail consumers, due to the high demand for professional needs, especially for aesthetic suggestions, tourism retail consumers are easy to rely on professional salespeople, thus to a certain extent affecting perceived value, more affecting satisfaction, and loyalty.

From the relevant research of this study, it can be found that improving the quality of sales personnel, service awareness, and paying attention to the emotional changes of tourism retail consumers will create a positive shopping behavior of tourism retail consumers, establish a good interactive relationship with tourism retail consumers, and improve the satisfaction of tourism retail consumers (Na, 2017).

Sales personnel provide service contact process for consumers. If they can provide professional services with a certain image design angle, tourism retail consumers can not only feel the concept and culture of the brand but also have a special interest in outlet shopping, to improve the evaluation of outlet shopping.

(3) Establish good after-sales relationships and increase trust relationships

The key groups of after-sales service are regardless of age, education, and gender, so this is more difficult to maintain, but every customer who goes out of outlets may need such follow-up services to them protect your rights (Xuecheng et al., 2016). If it is said that pre-sales marketing and promotion in the face of outlets, then after-sales is the mouth of outlets to communicate with customers. If the tourism retail consumers are slow in this aspect, the service of outlets will bring some negative effects soon, and the customer flow and public praise that they have worked hard to obtain will be lost in 1 day.

In the process of sales, it is necessary to emphasize the professionalism of sales staff as in the ordinary urban stores (Yi et al., 2014). To see the after-sales department as important as the pre-sales department is to be responsible for the future of the outlets itself. For example, many luxury goods have after-sales attitudes such as “lifetime warranty” and “problem replacement” that also need to be inherited in outlets consumption.

No matter how the perceived value of customers is, customer satisfaction has no significant difference with each variable. Therefore, how to grasp customer satisfaction and do a good job in after-sales service, the satisfaction will be greatly improved, so the synchronization of a performance and after-sales service is also a way of operation. This point not only exists for outlets’ managers but also needs to be conveyed to the frontline sales staff.

### Suggestions for Outlets’ Managers

(1) Pay attention to the voice of the Outlets Management Committee and strengthen the awareness of tourism retail

The tourism industry is of great importance to society. In the daily management and operation of outlets, there are three basic organizations: outlets’ management, brand side, and brand agent.

If the participation of outlets’ managers is not enough, the planning of outlets will be out of order; if the brand side lack supervision on authorization, the experience of outlets will be inconsistent with that of the flagship store of the center. It is necessary to attach importance to the integration of the three parties, establish the outlet’s management committee, and conduct benign circle management (Kent et al., 1983).

In terms of brand management, it is necessary to emphasize the right to speak of the management of brand licensee, eliminate brands and agents that are not in conformity with the joint venture quality, and multi-parties participate in retail tourism planning of outlets area.

The special construction awareness of tourism retail can be realized through the layout of outlets’ management. Most outlets are connected with the scenic spot and far away from the urban area. Therefore, it is necessary to carry out unified planning for the overall food, accommodation, and transportation, and accelerate the construction of tourism infrastructure, so that travelers can feel a closer connection between the outlet and the scenic spot, to better enhance the perceived value (Kim et al., 2011).

Pay attention to the promotion of high-level shopping connotation to better meet the increasingly diverse motivations of tourists. It is necessary to maintain the strength, such as the appearance of the local characteristics of tourism decoration, modern functional facilities, providing local cuisine, and other ways to attract tourism retail consumers (Moon and Han, 2019).

For example, Japan Gotemba outlets, with traditional Japanese architecture as its entry style, provide a viewing platform to enjoy the beautiful scenery of Mount Fuji, provides local Matcha tea tasting service, and Japanese Geisha performance, so that people can feel the original Japanese style.

As an important part of tourism shopping, outlets are not only a shopping place but also space for people to travel and entertain (Na, 2017). Therefore, a more sophisticated design can bring a more rich consumption experience to tourism retail consumers, which can meet the multi-dimensional experience needs of tourism retail consumers, to enhance the perceived value of tourism retail consumers. To provide tourism retail consumers with a high level of cultural enjoyment, further enhance the highlights and characteristics of outlets, to achieve a more effective target of attracting tourists.

(2) Strengthen experience marketing and enhance the perceived value of tourism retail customers

As an international brand from Europe, outlets should consider the principle of rapidly expanding the number of stores based on the localization strategy, and increasing the perceived value of tourism retail customers, to win the loyalty and satisfaction of tourism retail consumption in the way of experiential marketing.

The perceived value of tourism retail customers directly affects whether they are satisfied with their consumption. Therefore, to improve their consumption satisfaction, it is necessary to improve their perceived value as much as possible.

Through factor analysis, this study verifies the four dimensions of perceived value, namely cost value, emotional value, social value, and functional value. These aspects are also needed to enhance the perceived value of travel retail customers. However, these factors are not independent individuals, they are interrelated with each other, and cannot be separated for research. It is reflected in the following special aspects.

(a) Experience-oriented transformation

In the outlets’ shopping centers, driven by inner needs, tourists not only pursue high cost-effective emotional experience but also hope to relieve daily pressure through tourism and increase the time spent with relatives and friends. Consumers are attracted by outlets’ appearance construction, local food, rich activities, and other aspects to participate in outlets’ shopping consumption. The research further verifies that outlets’ emotional marketing has a significant impact on satisfaction and loyalty.

Outlets’ management should attach importance to and speed up the transformation, change the single marketing means, should not treat outlets only as a consumption machine, but should build them into an experiential place. Different from the traditional concept of using all the space as far as possible to obtain economic benefits, outlets should carry out more reasonable public space planning, and bring the tourism retail consumers an all-round good experience of vision, hearing, smell, touch, and other senses through innovative design.

Through novel and interesting design and environmental architectural style related to the scenic spot, outlets bring more experiences beyond shopping to the tourism retail consumers. With the characteristic rest place, the tourism retail consumers can satisfy the desire of taking photos while resting.

(b) The setting of shopping experience scenes for female groups

Outlets need to adjust to the needs of women, such as women’s day, mother’s day, valentine’s day, and other festivals, and outlets should set up the corresponding women’s service green channel. In terms of the outlet’s service design, women’s demand, such as the establishment of an independent cosmetics room in the toilet, is also one of the outlet’s functional values, which needs to be considered and improved.

Outlets should give full consideration to the individual differences of tourism retail consumers, take into account the needs of different age, gender, and income groups in product layout planning, architectural design, and construction as much as possible, and consider that women are the main group of outlet shopping. In this way, it is more beneficial to enhance the perceived value of tourism retail customers, and then improve the satisfaction and loyalty of tourism retail consumers.

(c) Emphasis on the shopping experience of elderly groups

The elderly are an idle social network group. Many marketing programs think that the elderly have insufficient purchasing power and cannot travel. Old people have plenty of time. In their later years, they often stay with their grandchildren or go out for a walk-in group. When they are not alone, the surrounding groups will have purchasing power if they are children. If it is a group of friends, a person will often buy together.

The information obtained by the elderly is easier to spread, which is equivalent to that obtained by the whole family. The establishment of experiential marketing targeted at the social life of such groups can broaden outlet shopping groups and access to basic customers, thus facilitating the realization of outlets’ operation plan.

(3) Strictly control the brand qualification of outlets

To improve the customer perceived value of tourism retail, the quality of products and services should be guaranteed at first. When tourism retail consumers feel that the benefits brought by-products or services exceed the cost of purchase, they will be willing to buy such products or services.

For outlets, on the one hand, it is necessary to strictly monitor the brands and products settled in outlets; on the other hand, it can also allow agents to directly participate in the creation and design of products and buildings, and to brainstorm ideas so that travel retail consumers could provide their insights. Not only can travel retail consumers feel valued and respected, but outlets can also get more creativity and ideas from travel retail consumers (Xuecheng et al., 2016).

If outlets want to keep a foothold on the market and get the recognition of tourism retail consumers, they must change the traditional sales concept and method, strictly control the settled brands, pay attention to the rise of more social influencers’ light luxury brands, including the trendy brand varieties loved by young groups, to meet the needs of more tourism retail consumers and seek a broader development space (Yi et al., 2014).

(4) Accurately locate customer group portraits

Online shopping platforms, according to different types of people, position the type of people they belong to, provide corresponding products, and develop the consumer psychology of continuous shopping.

The survey shows that there is no big problem in the purchasing power of tourists who choose to travel abroad. With the diversification of social intercourse forms and the expansion of publicity channels, more and more people consume rationally. Meanwhile, they think that an outlet is an entertainment place. According to big data, the number of people who take consumption shopping as a kind of decompression interest has exceeded 70%. With the development and improvement of artificial intelligence technology and maturity, outlets’ managers can use a clustering method to analyze the proportion of each month’s members’ bills in outlets and match them with their personal information. Then, in the form of SMS or e-mail, we will create the color pages of the current month or season for the outlets’ activities, which will be targeted to different participants, such as salary section, academic work, age, and other key elements.

The data returned is not only a basis for making promotional materials to improve customer loyalty but also an important basis for outlets to choose the method and strength of monthly discount products. According to the path factor of the above model, outlets’ managers can predict the result of this approach. First, the tourism retail consumption loyalty will be greatly improved, and then the functional value, emotional value, cost value, and social value will also be improved for customers, which fully indicates that outlets’ activities have an important role in improving their services.

With the rapid development of the virtual economy and the bottleneck of offline real business, outlets are facing more and more challenges and competitive pressures, and the development situation is not optimistic. For the long-term tourism retail consumers, outlets’ managers should increase the management of online shopping, as well as the entry criteria of online shopping, so that after the end of the travel, the tourism retail consumers can continue to carry out outlet shopping through the Internet, mobile phones, and other ways.

(5) Strengthen feedback management

Outlets should actively collect and return the opinions of tourism retail consumers. It can use the ways of prize seeking, questionnaire survey, random interview, and online interaction to understand the real feelings of tourism retail consumers, so that the collected opinions can truly reflect the inner feelings of tourism retail consumers.

After collecting opinions, outlets’ managers should improve the feedback from retail tourism consumers, they also need to make a return visit to ask tourism retail consumers if there is any improvement in outlets after feedback. Second, the managers should collate the experience feedback of tourism retail consumers, set up a perfect database, classify the feedback according to the priority, and carry out the improvement one by one. For the significant contribution of tourism retail, consumers can be given a certain reward to encourage more tourism retail consumers to provide valuable advice.

Outlets’ managers should strengthen communication with travel retail consumers, make full use of current advanced Internet resources, improve communication channels with travel retail consumers, allow travel retail consumers to speak freely on the platform, and promptly answer the questions of travel retail consumers and meet the needs of travel retail consumers. Change the goal from pursuing sales volume to pursuing higher satisfaction of travel retail consumers, so that they can provide higher quality services to travel retail consumers and obtain higher satisfaction and loyalty of travel retail consumers.

With the global development of tourism retail, the relationship between outlets and scenic spots is becoming more and more diversified. The former single-purpose sales behavior has gradually changed, while the consumption motivation of tourism retail consumers has shown diversification. To strengthen the experience marketing, there is a need to strengthen both personal experience marketing and social experience marketing, which can enhance the perceived value of tourism retail consumers.

## Data Availability Statement

The raw data supporting the conclusions of this article will be made available by the authors, without undue reservation.

## Author Contributions

All authors listed have made a substantial, direct, and intellectual contribution to the work, and approved it for publication.

## Conflict of Interest

The authors declare that the research was conducted in the absence of any commercial or financial relationships that could be construed as a potential conflict of interest.

## Publisher’s Note

All claims expressed in this article are solely those of the authors and do not necessarily represent those of their affiliated organizations, or those of the publisher, the editors and the reviewers. Any product that may be evaluated in this article, or claim that may be made by its manufacturer, is not guaranteed or endorsed by the publisher.
